# Design considerations of benchtop fluid flow bioreactors for bio-engineered tissue equivalents *in vitro*

**DOI:** 10.1016/j.bbiosy.2022.100063

**Published:** 2022-08-31

**Authors:** H.W. Hoyle, C.M.L. Stenger, S.A. Przyborski

**Affiliations:** a Department of Biosciences, Durham University, South Road, Durham DH1 3LE, UK; b NETPark Incubator, Reprocell Europe Ltd., Thomas Wright Way, Sedgefield TS21 3FD, UK

**Keywords:** Bioreactors, Tissue engineering, Fluid flow, Cell culture, 3D, three-dimensional, ABS, acrylonitrile butadiene styrene, ALI, air-liquid interface, CFD, computational fluid dynamics, ECM, extracellular matrix, FDM, fused deposition modelling, PC, polycarbonate, PET, polyethylene terephthalate, PLA, polylactic acid, PTFE, polytetrafluoroethylene, SLA, stereolithography, UL, unstirred layer, UV, ultraviolet light

## Abstract

One of the major aims of bio-engineering tissue equivalents *in vitro* is to create physiologically relevant culture conditions to accurately recreate the cellular microenvironment. This often includes incorporation of factors such as the extracellular matrix, co-culture of multiple cell types and three-dimensional culture techniques. These advanced techniques can recapitulate some of the properties of tissue *in vivo*, however fluid flow is a key aspect that is often absent. Fluid flow can be introduced into cell and tissue culture using bioreactors, which are becoming increasingly common as we seek to produce increasingly accurate tissue models. Bespoke technology is continuously being developed to tailor systems for specific applications and to allow compatibility with a range of culture techniques. For effective perfusion of a tissue culture many parameters can be controlled, ranging from impacts of the fluid flow such as increased shear stress and mass transport, to potentially unwanted side effects such as temperature fluctuations. A thorough understanding of these properties and their implications on the culture model can aid with a more accurate interpretation of results. Improved and more complete characterisation of bioreactor properties will also lead to greater accuracy when reporting culture conditions in protocols, aiding experimental reproducibility, and allowing more precise comparison of results between different systems. In this review we provide an analysis of the different factors involved in the development of benchtop flow bioreactors and their potential biological impacts across a range of applications.

## Highlights

• Bioreactors are being widely developed and used for tissue engineering.
• Introducing convective fluid flow in cell culture can boost physiological relevance.
• The outcome of a culture is influenced by fluid flow and apparatus-specific factors.
• Choice of design parameters allow tailoring of apparatus for specific applications.
• Future development is key to improving performance of bioengineered tissues.

## 1 Introduction

**Fig. 1:**
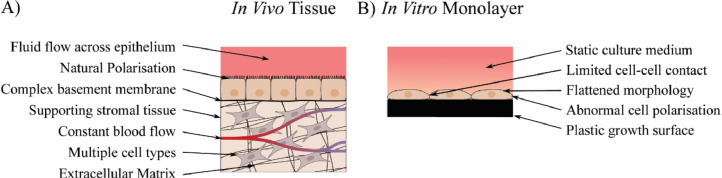
The cellular microenvironment *in vivo* and *in vitro*. (A) In the body, cells exist in a complex microenvironment surrounded by a wide range of stimuli (an example of an epithelial tissue is shown). These include mechanical stimulation such as from blood flow, contact between a large number of cells, both of the same type and different types, and contact with structural support such as the extracellular matrix and basement membranes. (B) Many of these factors are missing in standard *in vitro* monolayer culture. The lack of a 3D growth surface leads cells to flatten out, minimising contact with other cells. Cells adhere to the growth surface in a single direction, leading to an enforced polarisation which may not resemble that found *in vivo*.

The microenvironment of cells *in vivo* is a highly complex and dynamic system which is regulated by a number of factors (Fig. 1). These factors have a significant impact on the structure and function of cells and therefore are key to the successful creation of tissue models, though are often absent in conventional *in vitro* culture methods. Techniques such as three-dimensional (3D) cell culture can be used to provide a more natural morphology for the cells [1,2], and often incorporate other components such as extracellular matrix and growth factors which can further aid in the formation of functional tissue models [3]. Co-culture of multiple cell types is also a commonly used technique, possible in either a paracrine or juxtacrine manner, to incorporate native tissue interactions such as that between stromal supports and epithelia which can lead to enhanced polarisation and functionality in epithelial cells [4,5], or between immune cells and the surrounding tissue to model the effects of complex pathways such as diseases and drug responses [6], [7], [8].

By combining these methods, complex tissue equivalents can be generated for modelling a wide range of organs and tissues. These vary in complexity from utilising single cell types in a 3D structure to co-culture of several cell types and incorporation of physiologically relevant ECM. Full thickness models of the skin are a widely used example of a tissue model, due to the ethical concerns with animal models as well as the European Union ban on testing cosmetics on animals in 2013 [9]. These incorporate a stratified epidermis and supporting dermal compartment to allow interplay between the keratinocytes and fibroblasts [10]. Further complexity can be created through the inclusion of cell types such as melanocytes, Langerhans cells and endothelial cells, making it possible to model processes such as UV-induced tissue damage and investigate potential treatments [11,12]. Tissue equivalents have also been created for internal organs such as the lungs [13], kidneys [14], [15], [16], liver [17,18] and intestines [4,19,20]. Testing these models with drugs has been frequently performed and results often demonstrate an improved predictive response, though variable between different models, compared to 2D cultures [21,22]. Other factors such as the use of cell lines instead of primary cells can also significantly alter the outcomes of such experiments, making comparisons between studies more difficult [23,24]. Despite the potential sources of variation between these models, tissue equivalents represent an improvement over standard 2D cell cultures and are a step closer to accurately recapitulating the *in vivo* microenvironment [25].

These techniques, whilst able to recreate aspects of the *in vivo* microenvironment, often utilise a static culture medium which lacks many of the dynamic cues created by the movement of fluids in tissue. To address this shortfall, fluid flow may be introduced when culturing cell and tissue models using bioreactor technologies. These allow the creation of the dynamic conditions found in native tissue, such as blood and interstitial fluid flow. This can not only provide mechanical stimulation of cells, but also allows the levels of nutrients and metabolites in the medium to be maintained at more consistent levels, reducing the variability caused by factors such as medium changes and unstirred layers [26].

A wide variety of methods can be used to drive the fluid flow in a bioreactor, such as through the use of pumps or rotary motion. The level of complexity can also be hugely variable depending on the desired level of control, the tissue complexity, and the requirements for outputs such as real-time data readouts. This complexity brings with it many properties which need to be understood to ensure the systems reach their full potential from both the biological and engineering perspectives, and to make sure that data is correctly interpreted. Combined with the fact that the field of tissue engineering is highly interdisciplinary and requires researchers to have a broad knowledge of many fields means that there is potential for knowledge gaps which can be a hindrance to the development of effective bioreactor systems. Drawing this information together to give abroad overview of the different properties which may be encountered could therefore aid in the effective development of future bioreactors.

**Fig. 2:**
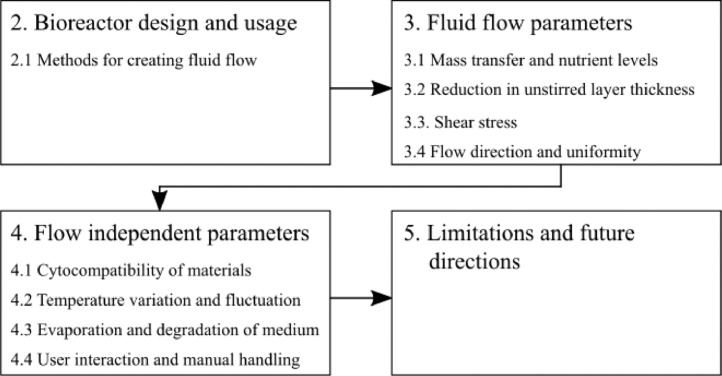
An overview of the parameters presented in this review. Section 2 covers the different techniques for creating fluid flow. In Section 3 the different parameters which are affected by fluid flow are explored, followed by the parameters which are independent of the fluid flow in Section 4. Finally the limitations of current techniques and future directions will be discussed in Section 5.

In this review we provide an overview of the parameters involved in the creation of bioreactor technology to support cell and tissue growth along with the impacts these factors can have on cultured cells and tissues. An outline of these parameters is shown in Fig. 2. In Section 2, the different types of bioreactor design and ways in which they have been used in literature will be discussed. In Section 3, different culture properties which are influenced by the fluid flow will be described. Section 4 will work through a series of other culture properties which are not affected by fluid flow, however, can be influenced by design choices such as the geometry of the bioreactor system. Finally, in Section 5 the limitations of current methods and future directions will be discussed. A thorough understanding of these factors can have the potential to help explain some unexpected results and aid with creation of a biological system which represents *in vivo* tissue accurately and effectively. The properties to be discussed in this review can be separated into two major categories: Fluid flow parameters and flow-independent properties. Within these two categories are different properties which will be discussed throughout this review.

## 2 Bioreactor design and usage

**Fig. 3:**
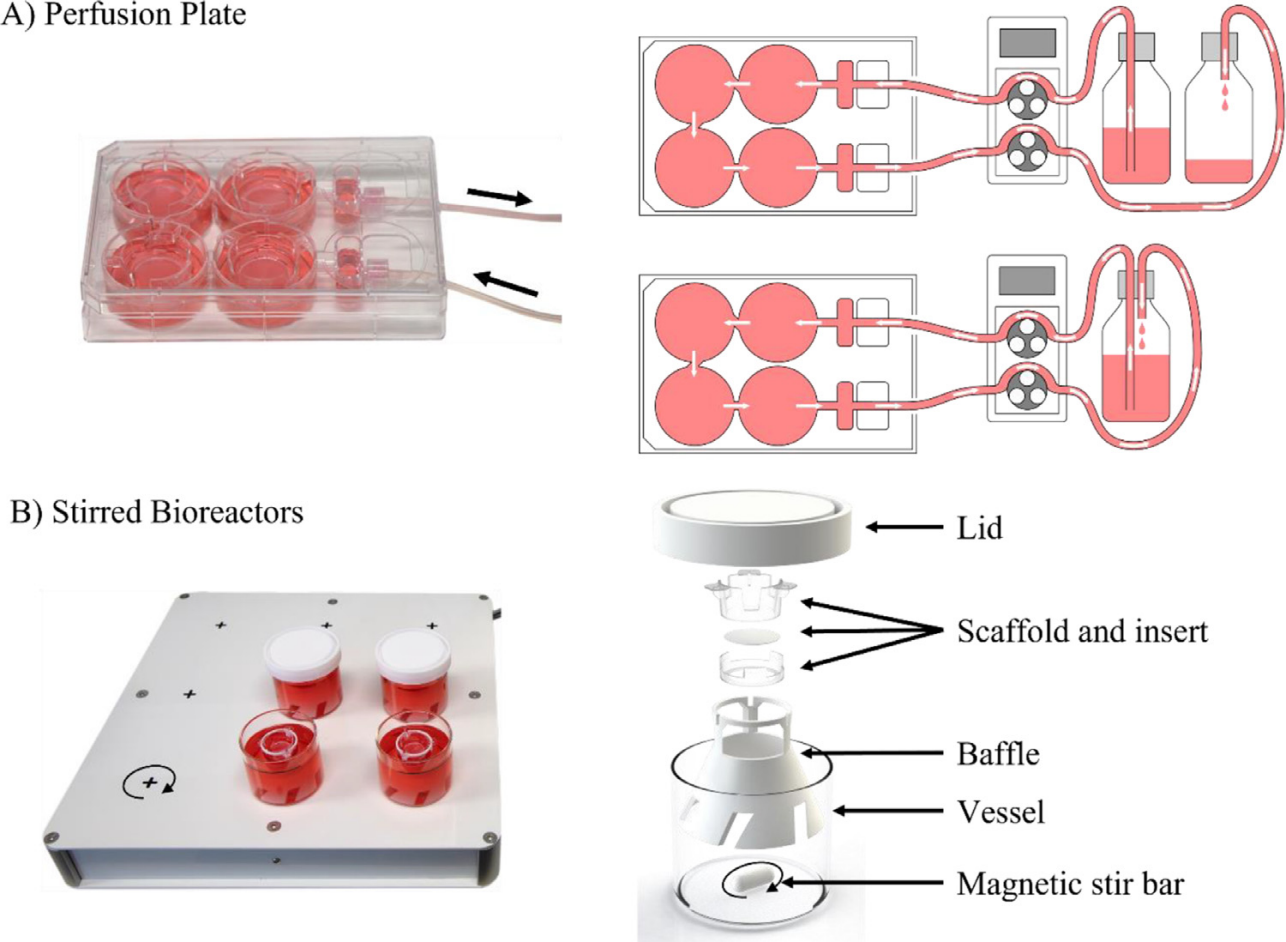
Fluid flow in cell culture inserts can be introduced in different ways. (A) A modified culture plate which allows medium to be pumped between 4 wells housing a scaffold insert. Additional equipment required includes a peristaltic pump and fresh and waste medium reservoirs. The inserts can be cultured in this system in the same way as with a standard 6-well plate. (B) A stirred bioreactor system for introducing fluid flow to scaffold inserts. This utilises a magnetic stirrer and uses a baffle to help direct the flow. A larger volume of medium is used to minimise the need for regular replenishment.

Bioreactors are increasingly used in cell culture for the creation of tissue equivalents in dynamic conditions with physiologically relevant fluid flow. These can range widely in complexity and utilise a range of additional components to generate the desired conditions. The main part of a bioreactor is the vessel in which the culture is held. This can be a simple, readily available piece of culture plasticware such as a multi-well plate or can be a bespoke vessel with complex control systems designed to direct fluid flow and perfusion of the tissue construct. The use of different three-dimensional cell culture techniques such as hydrogels and scaffolds in these systems is common. For example, a simple porous polystyrene scaffold used in 3D cell culture [27] is held in a cell culture insert and can be placed in a standard cell culture multi-well plate for static culture. Fig. 3 shows examples of two different designs that have been developed to maintain these 3D scaffold cultures in inserts whilst also providing fluid flow. These different techniques introduce fluid flow and of different types, demonstrating that flexibility that can be built into dynamic culture systems. A standard 6-well plate allows both submerged and air-liquid interface culture to be performed with ease. The perfusion plate shown in Fig. 3A utilises pumped flow through the wells in a continuous-fed manner to provide fluid flow across the 3D scaffold culture [28]. This allows inserts to be cultured in the same manner as a standard 6-well plate, minimising the variables which are changed. The rate of fluid flow is controlled through the pump whilst medium can be either recirculated or continually refreshed. The bioreactor system in Fig. 3B uses a batch-fed stirred tank system which can draw fluid both across and through the membrane [29]. This uses a large volume of batch-fed medium to minimise the need for additional changes, and the fluid flow rate is controlled by the stir speed. These demonstrate the flexibility of bioreactor systems for providing fluid flow with a range of properties and using different methods whilst able to support a standard culture insert. There are many alternative methods which can also be used to introduce fluid flow, some of which will be highlighted in the next section.

### 2.1 Methods used for creating fluid flow

**Fig. 4:**
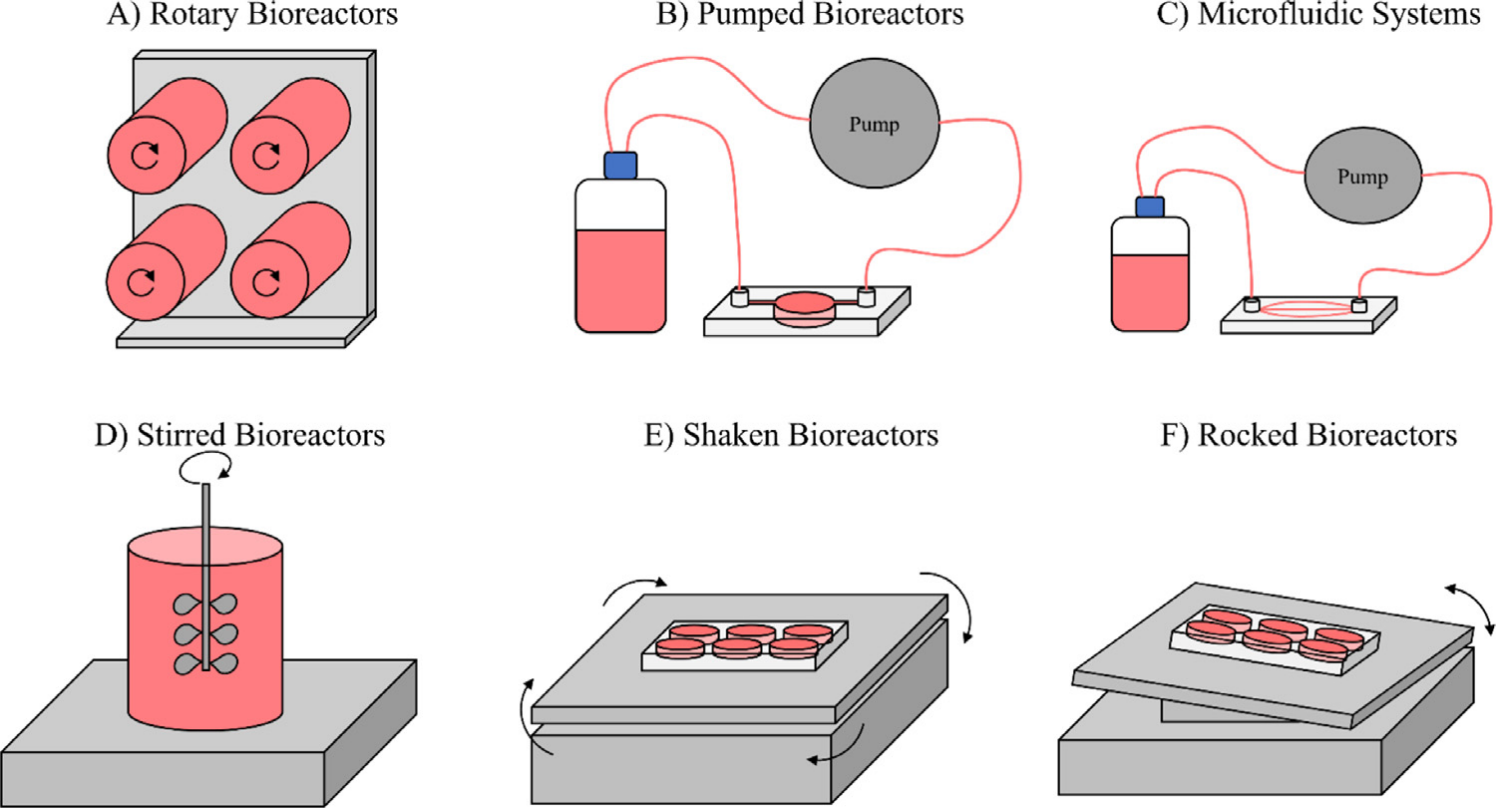
Commonly used technologies to introduce fluid flow into cell culture. Different techniques lead to flow patterns which can have desirable properties for modelling different tissues *in vitro.* The designs shown here are simple methods for creating fluid movement in cell culture. Variations exist for each design which vary widely in complexity. Simple systems may use conventional cell culture plasticware on equipment such as a mechanical rocker to produce fluid flow whilst maximising ease of use. More complex designs combine several of these technologies to maximise physiological relevance.

A variety of techniques can be used to introduce fluid flow into a cell culture system, with a range of commonly used approaches shown in Fig. 4. These methods can be used individually, or multiple methods can be incorporated into one system to give a higher level of control over the conditions. Further apparatus can be added including heat exchanges, humidifiers, bubble traps and oxygenators, which may be needed with some bioreactors if specific culture conditions are required. A range of monitoring systems can also be incorporated to measure properties such as pH, temperature, and oxygenation and provide insight into conditions within the bioreactor. The more complex apparatus typically has a trade-off in terms of cost, spatial requirements, and technical ability.

There are a range of commercially available bioreactor systems available for cell and tissue culture (Table 1). These also have a range of scales, from microfluidic chips to vessels which can hold litres of medium. There are also variations in complexity, from simple vessels with a minimal system to generate fluid flow to bioreactors which can monitor atmospheric conditions [30,31] or culture properties such as trans-epithelial electrical resistance of epithelial barriers [32].

**Table 1:** Commercially available bioreactor systems for cell and tissue culture under fluid flow conditions. These table has a representation of different systems and is not exhaustive, with a wide range of different systems available for specific applications. ALI = Air-Liquid Interface, ECM = Extracellular Matrix.

Manufacturer	System	Bioreactor Type	Set-up	Volume per culture	Basic Control Functions	Cell location	Refs.
Synthecon	Rotary Cell Culture System	Rotary	Single, dual, or quad rotary vessels	Customizable	Rotation speed	In suspension or on microcarriers	[33]
CelVivo	ClinoStar™ / ClinoReactor™	Rotary	Six rotary vessels in a stand-alone incubator	10 mL	Rotation Speed Temperature CO_2_ Level	In suspension or on microcarriers	[30]
Reprocell	Alvetex® Perfusion Plate	Pumped	Plate with 4 interconnected wells	10 mL	Flow rate	In scaffold, submerged or at ALI	[34]
Kirkstall	Quasi Vivo®	Pumped / Microfluidic	Single or multiple connected chambers	2 - 4 mL	Flow rate	In scaffold, submerged or at ALI, barrier model option	[35]
ibidi	Pump System	Pumped / Microfluidic	Microslides with chambers	60 µL	Flow rate	Channel surface	[36]
Emulate	Human Emulation System	Pumped / Microfluidic	Stretchable dual channel microchips with a porous membrane	15 - 35 µL	Flow rate Cyclic stretch	Porous membrane, channel surface	[31]
ABLE® Biott®	Magnetic Stir System	Stirred	Impeller stirred bioreactors	5 −100 mL	Stir speed	In suspension or on microcarriers	[37]
Corning™	Spinner Flasks	Stirred	Impeller stirred bioreactors	125 mL – 36 L	Stir speed	In suspension or on microcarriers	[38]
Mimetas	OrganoFlow® / OrganoPlate®	Rocked / Microfluidic	Multichannel microchips with oscillatory perfusion	50 µL	Rocker speed Rocker angle	On channel surface, on or in ECM	[39]

Although many studies utilise bespoke bioreactor systems, the use of commercially available bioreactors has a number of advantages. Key amongst these is the reproducibility benefit, with laboratories around the world able to access these systems to carry out experiments. There has also been a large amount of characterisation already performed by different research groups for some of these bioreactors, and often literature is available for applications of interest, providing a starting point to build upon rather than having to perform full optimisation before using the systems.

Rotary bioreactors (Fig. 4A), such as the Rotary Cell Culture System produced by Synthecon [33] and the ClinoReactor™ by CelVivo [30], allow the formation of aggregate cultures in a dynamic environment across a range of volumes. The speed of rotation allows for control over mass transfer and shear stress, as well as giving control over the average size of the aggregates formed [40,41]. Further complexity can be incorporated into the cultures using materials such as the use of microcarriers [42,43] and scaffolds [44] to provide extracellular matrix components and give additional spatial control over tissue structures. On top of their simplicity, these systems can also be readily scaled between millilitre and litre volumes, with both single use and autoclavable vessels available at a range of sizes. Additional features such as atmospheric control and real-time imaging of cultures are also available with some systems [30].

Pumped bioreactors (Fig. 4B) can be designed to use existing culture ware or made as bespoke systems. These utilise a pump, often peristaltic, to provide a flow of medium across a culture. These also usually employ either one or two reservoirs to provide a supply of medium, which allows either recirculation of the same medium or a constant supply of fresh medium. A wide range of different systems have been designed, incorporating different cell supports such as porous membranes [45], synthetic or extracellular matrix (ECM) scaffolds [46], [47], [48] and hydrogels [49,50]. An advantage of these systems is the ease of incorporating them into standard static 3D culture methods, allowing complex tissue equivalents to readily be perfused with medium without needing to simplify other aspects of the culture, as has been demonstrated with full-thickness skin equivalents [51,52]. These systems are available in a range of sizes, such as the 6-well plate format of the Alvetex® Perfusion Plate [34] or as microfluidic and organ-on-a-chip systems which require much smaller volumes of medium [31,35].

Microfluidic systems (Fig. 4C) are typically a subset of pumped bioreactors, utilising a fluidic chip to house the cell culture. These use much smaller volumes (microtiters) and flow rates, therefore presenting an effective system for fields such as drug discovery, where quantities of the test compounds may be limited [53]. A wide range of microfluidic chips are commercially available and production of custom chips using materials such as polydimethylsiloxane or polymethylmethacrylate can be performed with ease using several simple techniques [54]. These systems tend to be more sophisticated than other systems due to the need for a large number of components and tubing to connect them and the subsequent control requirements. Many opportunities are feasible using this technique such as linking multiple cultures together to create complex multi-cellular systems designed to simulate crosstalk between different tissues [55,56].

Stirred bioreactors (Fig. 4D) is a technique commonly found for producing larger quantities of cells and producing products such as antibodies or proteins but can also have utility for engineering cultured tissue models. Commercially available examples of these systems are commonly in the form of spinner flasks, such as the Able® Biott® and Corning™ systems [37,38]. These can come in a range of sizes to allow for scale-up production and typically use a larger volume of medium than other methods alongside batch feeding to maintain adequate nutrient levels across the culture period. The stir speed and shape of the stirrer allows for significant control over the mass transfer and shear stress in the system. Variation of the stir speed in this manner has been shown to impact the levels of different ECM components secreted by chondrocytes [57] and affect properties such as albumin secretion in HepG2 hepatocellular carcinoma cells [58]. One of the most common uses in tissue engineering is for the expansion of stem cells by maintaining stem cell pluripotency and offering an alternative to methods such as feeder layers [59], [60], [61]. This technique can also be used alongside 3D scaffold cultures to allow dynamic fluid flow to support tissue equivalents with a greater degree of structural control [62], [63], [64], [65].

Rocked and shaken bioreactors (Fig. 4E, F) are some of the simplest methods for creating fluid motion. These utilise low-cost equipment commonly found in the laboratory that can be housed within a cell culture incubator. They can be used in a variety of ways, such as with standard cell culture ware to provide additional mass transfer or bespoke plasticware can be designed for purposes such as allowing cultures to oscillate between contact with air and medium. These systems are also highly scalable due to the ability to stack multiple plates vertically without additional modification of the system. Pumpless organ-on-a-chip technology is also available in the form of the Mimetas OrganoFlow system [39]. This uses a rocker-based system to create fluid motion, removing the need for pumps and tubing and therefore requiring less complex apparatus. Studies have shown the utility of rocked perfusion to enhance mineral deposition with osteogenic progenitor cells [66,67], as well as for the maintenance of precision-cut liver slices through enhanced oxygen delivery [68,69]. Due to their simplicity and compatibility with standard culture plates, shaken bioreactors have been used in many studies to investigate the effects of shear stress on endothelial cells in 2D culture [70], [71], [72]. Some studies have also been demonstrated compatibility with Transwell® inserts, both using endothelial cells [73] and renal tubular epithelial cells [74]. One of the main limitations with this method is the lack of control over the direction of the fluid flow and the variability of the shear stress, with levels increasing with distance from the centre of the plate [75,76].

These different bioreactor designs demonstrate the range of ways that fluid flow can be introduced into cell and tissue culture, with the choice often dependant on cell type, 2D or 3D model requirements and the desired emulated physiological conditions. Suspension cells are commonly cultured in stirred or rotated systems, where the exact positioning of the cell relative to the fluid flow direction is not crucial. Instead, fluid flow is intended to facilitate mass transfer to support proliferation and aggregate formation. Selection of stirring speed is used to tune aggregate size and shape but is highly cell type and application dependant. Higher stirring speeds in spinner flask systems used for human induced pluripotent stem cells have been shown to form more homogenous round-shaped aggregates at higher stirring speeds without losing their pluripotency [77] whilst increasing the stirring speed too much can correlated to a reduction in aggregate size and increasing levels of cellular detachment from microcarriers [78,79]. As shear stress, velocity and pressure vary within stirred systems, shear stress values for suspension cells are not global but location dependant which should be taken in account when planning stirred suspension cultures [80]. A general assumption for suspension cells is that they can withstand high shear stress values, if they are naturally exposed to high flow rates *in vivo* like erythrocytes or leukocytes [81]. However, stem cells are often expanded in stirred systems and are more susceptible to shear stress, requiring fine tuning of the parameters to avoid loss of pluripotency [80].

Adherent cells require a substrate to grow on, which will affect perfusion characteristics. Although microcarriers for cell adhesion in stirred suspension cultures have been used, these systems do not allow for a tight control of a delicate microenvironment which is often necessary to archive desired tissue morphology and function. Adherent cells often exhibit polarization with a distinctive apical and basal side crucial for proper cell function and fluid flow affects this polarization [82]. Separation of these distinct compartments also allows for investigations into epithelial barrier and transport properties [83], studies which are of high value in areas such as intestinal drug absorption [84]. It is therefore favourable to use systems that allow for controlled flow regimes, like pumped or gravity driven systems with directed fluid flow. These forms of fluid flow allow for the incorporation of complex scaffolds and cellular supports, providing a basis for physiologically relevant recreations of tissue architecture [85]. Other cell type specific characteristics that should be taken into consideration when choosing a perfusion system, are metabolic activity, influencing how much medium is required, the frequency of medium changes and build-up of harmful metabolites as well as specific cues needed for certain differentiation processes such as an air-liquid interface for the differentiation of keratinocytes in models of the epidermis [86].

## 3 Fluid flow parameters

**Fig. 5:**
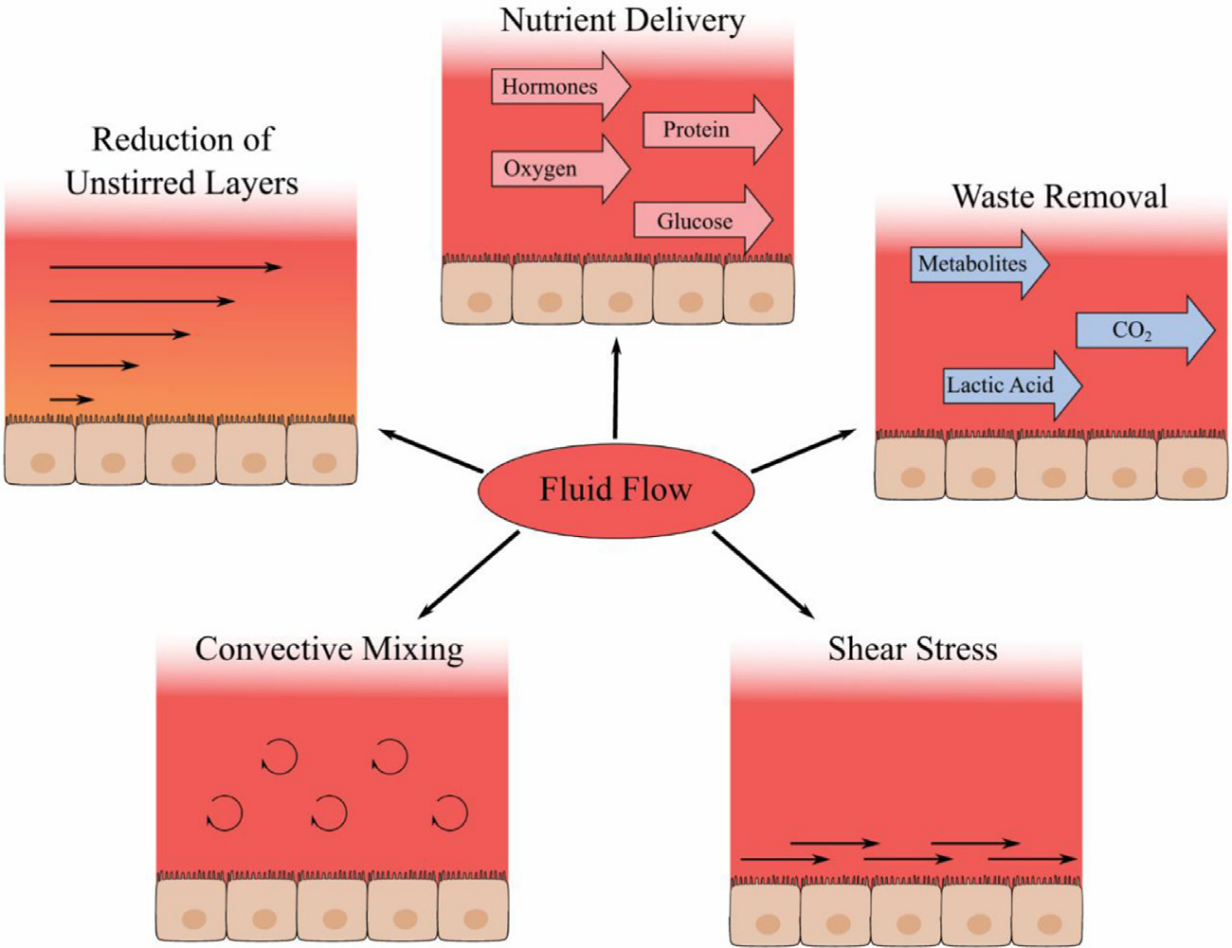
Direct impacts of fluid flow in cell culture. Fluid flow aids with healthy growth of cells through a variety of mechanisms. The supply of fresh nutrients, as well as the removal of metabolic waste can reduce nutritional stress on the cells. A reduction of the unstirred layer thickness and convective mixing aid with this process whilst also being useful for research such as transport studies. Shear stress presented to the cell surfaces creates mechanical signals which the cells respond to and can aid with maintaining physiological function however can have a detrimental effect at supraphysiological levels.

Bioreactor functions are centred around the controlled introduction of fluid flow to a cell or tissue culture. The addition of fluid flow has a major impact on a wide range of cell types with the exact properties of the fluid flow leading to different effects between varying cell types and tissues [87]. Some of the key impacts of fluid flow, as shown in Fig. 5, are on the mass transfer both in the form of nutrient delivery and waste removal [88,89], shear stress experienced by cells adjacent to the fluid flow [90,91], the convective mixing and resultant homogeneity of the media and the reduction in the size of the unstirred layers close to the cell boundary [92].

### 3.1 Mass transfer and nutrient levels

As cells take up media constituents such as glucose, proteins and vitamins for metabolic purposes, the levels of such molecules need to be replenished to maintain consistent culture conditions and healthy cells [93]. Supraphysiological levels of nutrients such as glucose, pyruvate and glutamine are often used for cell culture medium to allow for this reduction over time. While these concentrations were often derived empirically to maximise viability and proliferation, they do not necessarily mimic the environment *in vivo* and can induce further unwanted artifacts [94]. Many studies have demonstrated an induction of apoptosis in high glucose medium with a range of cell types [95], [96], [97], whilst improved medium formulations have been shown to produce more physiologically relevant metabolic levels and drug response in cancer cells [98], [99], [100]*.* On top of this, metabolic waste products such as lactic acid and ammonia can be damaging to cells and affect properties such as pH, with the levels of these building up over time in culture in the absence of frequent media changes [101].

Within bioreactors, motion of the fluid improves the level of mass transfer through convective mixing, on top of the diffusive mixing found in static culture. The mass transfer consists of the transport of both nutrients to tissue and of metabolites and waste products away from tissue [102]. Providing adequate mass transfer to cells can improve their proliferation, viability and function [26,103]. This is particularly relevant for the culture of stem cells which can have varied viability and differentiation states depending on nutrient availability [104] and for *ex vivo* tissue slices such as liver slices where high levels of oxygenation are typically required for long-term maintenance [105]. Effective mass transfer, both of nutrients to tissue and of metabolites and waste products away from tissue is therefore one of the key goals of fluid bioreactors for tissue engineering [102].

One of the most direct methods for increasing mass transfer is through an increase in fluid flow rate in a bioreactor. This can lead to an increase in convective mixing, as well as greater replenishment of medium around the cell surfaces therefore aiding mass transfer to and from the culture. Measurement of the relationship between fluid flow and mass transfer has been carried out for shaken bioreactors [106], impeller-based systems [107], [108], [109] however is rarely performed for bioreactors used in tissue engineering. The impact of fluid flow rates on cultures is often measured through cell viability or proliferation [110,111], though other changes can also be readily performed in some cases, such as albumin secretion in liver models [112,113] or the thickness and maturity of the epidermal barrier of skin models [114]. A beneficial impact typically tends to correlate with increasing flow rate until the point in which shear stress starts to have harmful impacts and outweighs the benefits of mass transfer, after which a decrease is measurable [111,112]. The impacts of shear stress within the system will be covered in more depth in Section 3.3.

Another factor which affects mass transfer is the geometry of a system. This can be beneficial or detrimental, depending on the bioreactor type and method of fluid movement. A key example of where the geometry can increase mass transfer is found in stirred bioreactor systems, in which the geometry of the impeller or stirrer has a major impact on the mass flow rate. The number of blades and blade angle, along with the impeller size, can lead to significant changes in mass transfer rates which in turn affects the performance of the cells within these systems [109,115,116]. Other geometric features which significantly alter the direction or rate of fluid flow, such as sharp corners, can lead to a shift from laminar flow towards turbulent flow. While generally regarded as less desirable in bioreactors than laminar flow, turbulent flow allows a higher level of convective mixing within the fluid layers. It can therefore be useful in regions of medium which are not in contact with cells, such as reservoirs, to maintain an even mix of molecules in the bulk medium.

The spatial position of cells also plays a role in the mass transfer and nutrient supply in a bioreactor system. Cells in direct contact with the fluid will see increased mass transfer whereas cells within the centre of a tissue mass are primarily supplied by diffusion through the tissue. Diffusion of oxygen is a relatively slow process and in tissue it has typically been measured to reach a maximum depth of between 100 and 200 µm from the vasculature and therefore presents a limiting factor for the size of tissue equivalents in the absence of vascularisation or fluid flow [117,118]. This is a problem commonly found in aggregates and spheroids, where interior regions suffer from a depletion of nutrients alongside a build-up of waste products, leading to a necrotic core [119,120]. Structures such as scaffolds or membranes used to support cells can also suffer from nutrient deficiency due to the reduced diffusion caused by the presence of the scaffold hence adequate mass transfer also plays an important role when using such materials [121]. The use of scaffolds with pores or channels to allow the perfusion of medium though the tissue construct can be an effective way to overcome this, with structural properties such as pore size and tortuosity impacting the mass transfer of solutes through the material [26,122]. Effective examples of this are hollow fibre bioreactors which use permeable fibres to transport medium through the culture. A precisely controlled distance between the interior cells and the flow of medium maintains effective mass transfer to all cells without limitations of diffusion distance. On top of this, the fibres also offer protection from shear stress, allowing high mass transport whilst minimising shear stress-related damage to cells [123,124].

#### 3.1.1 Oxygenation

One specific example of the importance of mass transfer and nutrient supply *in vitro* can be found in the case of oxygen. Oxygen is essential to maintain viable growth and function however there is a wide variability in the dissolved oxygen levels seen *in vivo,* both between different tissues and different cells within a tissue. Regions such as the peri‑sinusoidal bone marrow have been found to experience an oxygen tension as low as and 0.64 kPa [125] whilst on the other end of the spectrum renocortical tissue and periportal liver tissue can both have levels reaching around 8–10 kPa [126,127]. Cell culture medium in a 5% CO_2_, humidified incubator typically has a dissolved oxygen level of around 18–19 kPa, though this value is lower in the pericellular region due to the cellular depletion and has been found to be near zero for cells with high oxygen consumption such as hepatoma cell lines and renal epithelial cells [128,129]. Meanwhile, the value is around 13 kPa for arterial blood [130]. This demonstrates the large complexity of mimicking physiologically relevant oxygen concentrations *in vitro* and why there is not a “one size fits all” approach to bioreactor design.

**Fig. 6:**
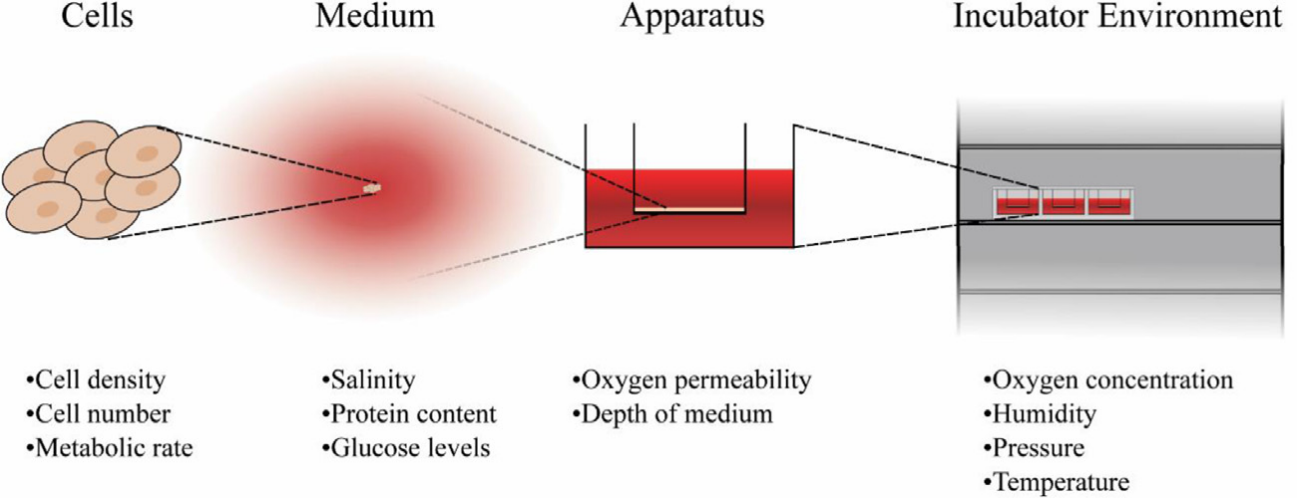
Properties which can impact the levels of dissolved oxygen in culture. The different properties can be divided by the scale upon which they act. At the smallest scale, the properties of the cells can change the oxygen properties through varying the rates of depletion in the surrounding media. Medium properties such as the molecular constituents have an impact through the changes in the oxygen solubility. Culture geometry such as the distance to the air-liquid interface and the permeability of vessel walls influence uptake into the medium. The atmosphere that the culture is in also has an impact, with factors such as humidity, pressure and temperature affecting local levels of oxygen concentration.

Fig. 6 shows some of the different properties of cell culture systems which can impact the oxygen received by cells. At the cellular level, factors such as cell density, quantity and metabolic rate all influence dissolved oxygen levels. Large quantities of cells or highly metabolic tissues deplete oxygen at a faster rate which may be higher than the diffusion rate of oxygen to the cells leading to reduced oxygen levels over time [131]. A second problem with large quantities of cells occurs when considering the diffusion distance of oxygen. As mentioned in the previous section, it is generally observed that the diffusion distance for oxygen within tissues is less than 200 µm. When creating tissues significantly larger than this a necrotic core is frequently reported and this distance therefore presents a size limitation for avascular *in vitro* tissues [132], [133], [134]. For medium, different formulations have different rates of oxygen solubility and diffusion depending on properties such as salinity and protein content [118]. The vessel used to hold cultures can lead to variability in oxygen levels due to factors such as the diffusion of oxygen through the vessel walls. This effect has been investigated for increasing the oxygen levels using highly gas-permeable materials in both static and dynamic culture systems [135], [136], [137], [138]. The depth of the culture within the medium also leads to changes in oxygen concentration, with the major limiting factor being the diffusion rate through the media, the further a culture is from the air the lower the dissolved oxygen levels will be. Atmospheric pressure and oxygen levels also impact the dissolution of oxygen into the media whilst aspects such as media depth and flow rate affect the diffusion and transport of oxygen through the media.

Additional oxygenation systems are often used in conjunction with perfusion bioreactors to achieve optimal conditions. These can range from placement of the bioreactor in an oxygenated incubator to self-contained oxygenation systems specific for the bioreactor [139]. Alternatively methods such as disruption of the gas-liquid boundary of the media has been used to increase oxygen concentration without an additional oxygen supply [140]. For applications which require lower oxygen levels a commonly used method is to reduce the partial pressure of atmospheric oxygen with technology such as hypoxic chambers. Alternatively reductions in oxygen delivery to media, either actively through the control of flow rates or passively through oxygen-permeable membranes, can be another useful tool particularly if access to a gas supply is limited [138,[141], [142], [143], [144]]. This leads to wide variability in the effectiveness of oxygenation systems and characterisation of oxygenation levels is important if results can be accurately replicated [131].

### 3.2 Reduction in unstirred layer thickness

**Fig. 7:**
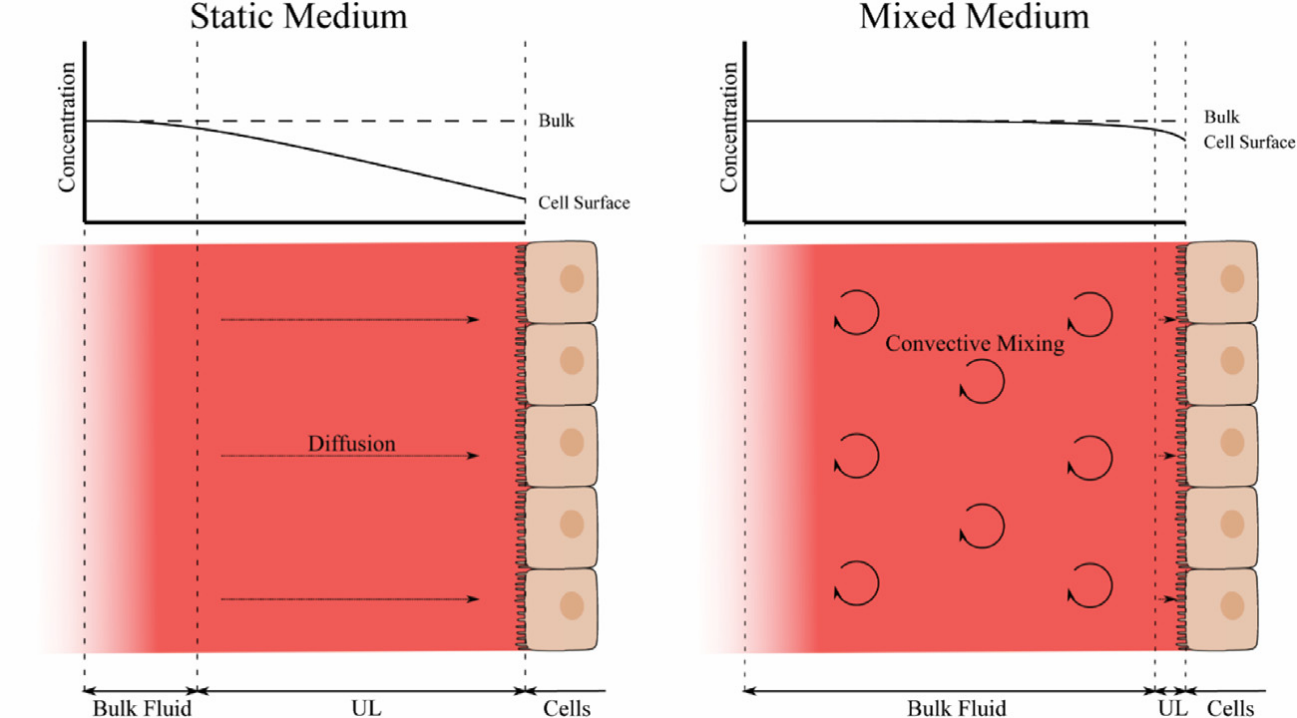
Unstirred layers in static and dynamic cell culture conditions. In static culture the medium is mixed through diffusion which can lead to a depletion in concentration close to the cell surface, a region termed the unstirred layer (UL). The application of convective mixing to the medium, such as with fluid flow, greatly increases the distribution of molecules within the medium and reduces the thickness of the unstirred layer, where diffusion is predominant. This in turn leads to greater levels of solute concentrations at the cell surface.

Unstirred water layers are regions close to membranes in which limited convective mixing occurs and so the majority of mixing occurs by diffusion of molecules [145]. These vary in size and impact depending on the particular solute, the viscosity of the fluid and any convective mixing that the fluid is undergoing. Fig. 7 shows the effect that a larger unstirred layer in static culture medium has on the solute concentration for nutrients at the cell surface compared to in mixed medium. The addition of convective mixing reduces the thickness of the unstirred layer and therefore increases the concentration at the cell surface. The impact on tissue culture is therefore unique to each individual tissue model and the exact impacts can be difficult to predict. The first potential problem caused by these is the diffusion of nutrients such as oxygen to the cells. Whilst uptake of oxygen into the cells depletes the concentration in these regions, the limited diffusion can lead to reduction in oxygen available to the cells and therefore lead to a greater level of oxygen stress than expected from the oxygen concentration in the bulk medium [146]. The size of the unstirred layers is generally regarded to be inversely proportional to the diffusion coefficient for a molecule [147]. Oxygen, with a high diffusion coefficient, can therefore be predicted to have a large unstirred layer with a thickness up to several hundred microns, as has been found for similar solutes such as carbon monoxide [148], and is therefore a far more likely rate-limiting step for uptake into cells than barriers such as the cell membrane.

As well as potential nutrient and metabolite diffusion problems, another example of an issue which can be caused by unstirred water layers is additional inaccuracy in measuring cellular transport for drug metabolism studies. The limitations to uptake caused by the unstirred layers can lead to a significant underprediction of uptake rates into cells. This can lead to errors in the measurement of membrane transport, but can also be problematic when calculating drug metabolism and clearance due to becoming the major rate limiting step [149,150]. These effects have been heavily studied in the context of gut permeability and hepatocyte drug clearance with results showing that perfusion or agitation of the medium can be an effective way to reduce the impact of unstirred layers on permeability measurements [151], [152], [153].

### 3.3 Shear stress

Shear stress is caused by the flow of fluid across a cell and is proportional to the fluid flow rate parallel to the cell surface. The level of shear stress is a key property of fluid flow in native tissue and can vary over many orders of magnitude between different regions in a tissue, such as blood or interstitial fluid. Cells can detect and respond to shear stresses, and therefore an accurate replication of *in vivo* shear stress levels can be beneficial for physiologically relevant function [154,155]. The mass transfer is also proportional to the fluid flow rate so this can lead to the difficult position of balancing a high mass transfer with the typically low levels of shear stress required for many cells [156]. The use of baffles, porous membranes and specific topographies to shield the cells from the bulk levels of fluid flow can be an effective way to maintain adequate mass transfer whilst keeping shear stress at a reasonable level [63,157,158].

*In vivo,* high shear stress is primarily experienced by endothelial cells lining blood vessels and these protect many of the other cell types from being directly in contact with the blood flow. The level of shear stress that endothelial cells receive varies hugely between different regions of vasculature with arterial endothelial cells experiencing levels around 1.5 Pa while in smaller vessels such as the liver sinusoids a range of around 5 to 10 mPa is experienced [159,160]. Factors such as variations in blood vessel diameter and bends in the vessels also contribute to further variation in the shear stress levels. Shear stress applied to endothelial cells at these levels has been shown to have some beneficial effects such as promoting angiogenesis and inhibiting inflammatory responses [161].

Due to the shielding function of the endothelial cells, most non-endothelial cells experience levels of shear stress which are many orders of magnitude lower and bioreactors often utilise shear stresses in the micropascal region [162,163]. Higher arterial values can still be useful with these cells, however, for researching the effects of mechanical stimulation and response to injury, for example mimicking damage to the endothelial wall and subsequent cellular responses [164], [165], [166]. Values of shear stress experienced by many cell types are difficult to acquire, either experimentally or computationally, due to the protection offered by the endothelial cells and therefore a range of shear stresses are often explored to experimentally determine the optimum values. Shear stress experienced by cells through interstitial fluid flow outside the vasculature has been the subject of numerous investigations, using *in vivo, in vitro* and *in silico* studies [166], [167], [168], [169], [170]. Depending on the application, extensive reviews are available which provide helpful compilations of experimentally acquired shear stress data for various cell types, particularly in the context of endothelial cells [164,[171], [172], [173]]. For example, a recent publication on organ-on-a-chip applications refers experimentally derived shear stress values for different cell types, which could be used as a starting point for initial experiments to empirically determine which levels of shear stress are beneficial for the desired outcome [174]. In the case of cell types with limited literature available, shear stress values from cell types residing in a similar environment to that being investigated could provide an initial estimation.

The liver, as an example with a high level of vasculature, is often used in fluid flow systems and many studies have performed this analysis to experimentally determine optimum conditions *in vitro* in the absence of accurate *in vivo* values [162,175,176]*.* Computational fluid dynamics (CFD) can be used alongside experimental methods to confirm that the levels of shear stress are within a similar range to estimated physiological levels, and this analysis has been performed previously for many commercial and bespoke bioreactor systems [177,178]. As well as shear stress*,* these simulations can be used to provide an insight into distribution and transport of nutrients such as oxygen, making them a powerful tool for understanding the complex conditions within a bioreactor system [179]. The ability to fine-tune the shear stress of a bioreactors in this way allows for accurate replication of *in vivo* conditions as well as expanding the possible applications and therefore increases the research value of a system [63,180,181]. While the use of CFD allows a bioreactor to be optimised for specific tissues, in practice this is not always performed as effectively as it could be. One recent review, for example, highlights that many bioreactors for bone tissue engineering are designed for generic bone tissue engineering, rather than for use modelling specific bones, which could therefore limit their utility due to the wide variability of conditions in different locations [182].

Viscosity of culture medium also has an impact on the shear stress experienced by cells under flow, however this is less well-studied compared to many of the factors mentioned previously. For the purposes of computational modelling, medium formulations are often assumed to have a viscosity close to that of water however addition of components such as proteins can lead to significantly increased viscosity. Addition of 10% foetal bovine serum, as is often used in cell culture, has been measured to cause an increase in the viscosity of the medium by 20–30% whilst further changes over time can also be caused by secretion of proteins from cells during culture [183]. The presence of serum can also cause the medium to have the non-Newtonian behaviour of shear thinning, adding further complexity to shear stress modelling through a reduction in viscosity at higher shear stresses [184].

### 3.4 Flow direction and uniformity

**Fig. 8:**
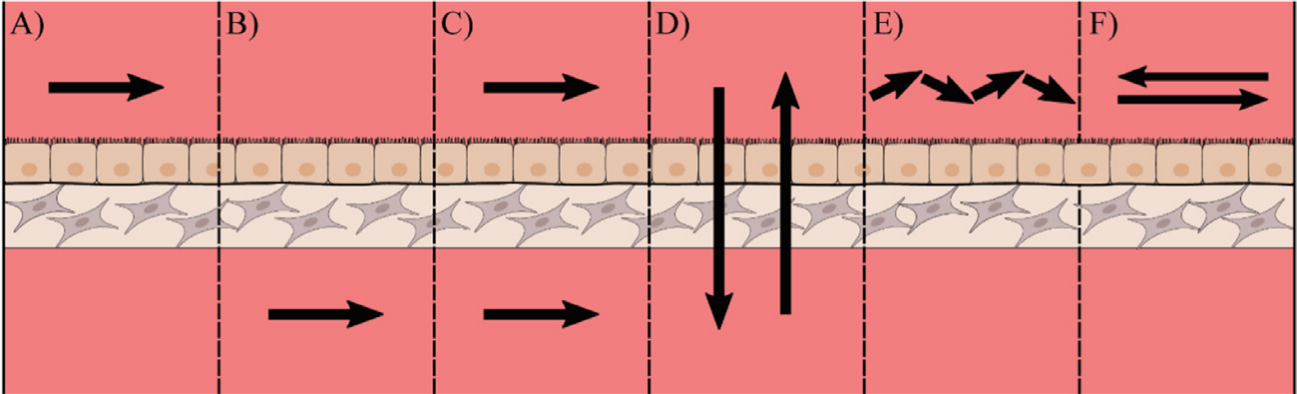
Methods for introducing fluid flow to an epithelial submucosal tissue model. With certain complex tissues consisting of an epithelial layer and a stromal compartment there are many possible ways to introduce flow. (A) Flow along the epithelial surface such as that found in the intestinal lumen (B) Flow along the stromal surface partially mimicking blood supply to a tissue (C) Flow across all surfaces of the model (D) Flow perfused through the tissue which can be used to improve the diffusion of nutrients such as oxygen to the centre of thicker tissue constructs. (E) Pulsatile flow, such as that produced by a peristaltic pump, can be used to mimic the pumping motion of the heart for modelling structures such as arteries. (F) Reciprocal oscillatory flow, which changes direction over a set period, allows convective mixing to be introduced using typically very simple apparatus such as a mechanical rocker.

The direction of the flow may need to be altered to cater for different tissues as illustrated in Fig. 4. The two most basic types of fluid flow are across the tissue or through the tissue and these can either be continuous or pulsatile. Flow across a tissue can be performed in specific compartments of a tissue: for example fluid flow over the epithelium (Fig. 8A) has been used for modelling intestinal epithelia [46]; perfusion below the stromal tissue (Fig. 8B) is widely used and has shown positive results for applications such as skin models and renal models [185,186]; flow all around a tissue can be useful for delivery of nutrients to tissues with high nutritional needs [187]. Flow through a tissue (Fig. 8D) can be more useful for perfusion of larger constructs as this method can be used to aid diffusion of molecules such as oxygen into the centre of these tissues [134,188].

Further increases to the complexity and physiological relevance of flow patterns can be the introduction of pulsatile or oscillatory flow. Pulsatile flow (Fig. 8E), such as that created by peristaltic pumps, is a more accurate representation of the flow of blood in regions such as larger arteries, replicating the conditions produced through the pumping mechanism of the heart. The addition of pulsatile flow to cultures can be straightforward to implement and has been shown to create beneficial conditions for arterial cell culture, with enhancements to endothelial cell function and angiogenesis [189], [190], [191]. As an alternative to pulsatile flow, reciprocal oscillatory flow can also be used, in which the flow alternates in direction over a designated period (Fig. 8F). Oscillatory flow patterns are highly relevant *in vivo*, especially in connective tissue, like bone tissue, and the vasculature. The effect of oscillatory flow perfusion regimes, either continuous, intermittent or varying in flow rate, has been studied for various cell types and tissue models. Intermittent oscillatory flow stimulations of mesenchymal stem cells during otherwise static conditions, have been shown to robustly upregulate osteogenic gene expression, drive lineage commitment and enhance collagen secretion and mineral deposition [192], [193], [194]. The application of continuous oscillatory flow regimes at low rates enhanced cell viability, uniform cell distribution and early osteogenesis in scaffold-supported osteoblast-like cell-based models [195,196]. In a combinatorial approach, bone-tissue models were continuously perfused at a low oscillatory rate with intermittent high-rate bouts to archive mechanical stimulation which significantly increased prostaglandin E_2_ levels, an important factor for bone formation [197].

Oscillatory flow can be induced using equipment such as a rocker creating a simple way to perfuse a model for media mixing whilst reducing the volume of addition equipment required. Simple oscillatory flow in this way has been used successfully for the differentiation of mesenchymal stem cells and the maintenance of precision-cut liver slices [67,68]. Modifications of these methods can also be used to create more complex systems for specific applications such as stacked substrates for patterned co-culture [198] and cultures with adjustable medium levels for raising cells to the air-liquid interface [199].

Complex geometries, in particular the presence of sharp angles, can lead to irregularities in the fluid flow and therefore mass transfer and shear stress [200], [201], [202], as well as leading to an increase in turbulence. Tissue culture bioreactors frequently utilise laminar flow, in which the motion of fluid particles is parallel to the bulk flow of the fluid, to create the desired conditions due to its uniformity and ease of modelling. Blood flow *in vivo* is also typically regarded as being laminar. Turbulent flow, which is more chaotic and disordered, leads to irregularities in the fluid flow and increases the complexity of the systems from a modelling perspective, though modern computational fluid dynamics software can be used to model these systems with relative ease [203]. Turbulent flow *in vivo* can be found in curved regions of the vasculature as well as at branch points and has been associated with increased risk of disease such as atherosclerosis [204,205]. The usefulness of turbulence within a tissue culture bioreactor is therefore dependant on the application, with turbulent regions effective for keeping the bulk medium mixed, though typically less desirable in regions of fluid that are in contact with cells due to the disruptive effects of the shear stress.

## 4 Flow-independent parameters

Aside from fluid flow parameters there are many other properties which can influence the outcomes of perfusion bioreactor techniques. Cytocompatibility issues can be caused when working with certain materials. The operation of a bioreactor and some design parameters can also cause deleterious effects such as fluctuations in culture temperature or a constant deviation from the desired temperature. Some feeding regimes can lead to issues such as media degradation, particularly if media changes are carried out infrequently as in some batch-fed systems. High levels of user interaction and manual handling can also be problematic by introducing additional variability to cultures which may impact reproducibility. Designing a bioreactor with an appreciation of these problems and with an aim to work around them as much as possible can lead to systems which more accurately agree with the theoretical outcomes of the system and are therefore of improved research value.

### 4.1 Cytocompatibility of materials

Routine cell culture ware has typically been made from materials which are known to have high cytocompatibility, with polystyrene being the most common material used for items such as multi-well plates and flasks. This is a material which is known to be very biologically inert and compatible with a wide range of cell types which makes it ideal for culture of cells with minimal side effects [206]. However other materials exist that have been thoroughly characterised for biocompatibility and may represent alternative options for use in bioreactor design [85,207,208].

Whilst polystyrene is a suitable material compatible with mass production techniques such as injection moulding, there are many other materials which are easier to work with at the scale of individual laboratories. Hard plastics such as polycarbonate (PC), polyethylene terephthalate (PET) and polytetrafluoroethylene (PTFE) are commonly used in bioreactors due to their ease of manufacture and minimal biological activity. PC and PET are regularly used as they have reasonable chemical resistance, high biocompatibility and can be readily treated to enhance cellular adherence [209,210]. This has led to many cell culture membranes, such as Transwell® and Millicell®, being made from these polymers. PTFE is highly inert and has a high melting temperature making it suitable for repeated autoclaving. It is a useful material for creating bioreactor components and is often used for the outer layer of magnetic stir bars. PTFE is also highly hydrophobic and has an extremely low coefficient of friction. This makes it useful in the case of moving parts to minimise wear, however it also means that PTFE requires further surface modification to be viable as a growth substrate for cells [211,212].

Rapid prototyping methods such as fused deposition modelling (FDM) or stereolithography (SLA) offer the ability to create custom designs within a short turnaround time. However, some of the materials typically used for these techniques are of limited value for cell culture. Acrylonitrile butadiene styrene (ABS) and MED610 have both been found to cause changes to cell viability and gene expression, such as an upregulation of oestrogen-response genes, in some cell lines, which shows that materials should be carefully chosen to ensure there are no specific biological effects with the cells of interest [213]. Polylactic acid (PLA) is another material which is widely used for FDM and has been shown by many studies to have a high level of biocompatibility [214,215]. Much like polystyrene used for conventional cell culture, PLA is a hydrophobic polymer and can benefit from surface modification to boost its adherent properties if cells are to be grown directly onto it [216].

A second problem found with techniques such as SLA is the leeching of compounds such as photoinitiators and plasticizers into the medium. Even with polymers which have been certified to be biocompatible, toxicity issues have been identified with cell culture. The continual release of photoinitiator into the medium for up to 7 days of culture has been demonstrated, along with a consequent reduction in viability [217,218]. In some cases these issues can be reduced by post-manufacture treatment with solvents such as ethanol [219].

Non-plastic materials are also often found in bioreactors. Glass and stainless steel are two common examples of these. Glass has been widely used for cell culture since its inception as both a growth surface and as a material for vessels. A high level of chemical resistance, minimal biological activity make glass a useful material for cell culture, and its high melting temperature makes it compatible with a range of heat sterilisation methods such as autoclaving and flaming, allowing glass components to be easily sterilised and reused. For this reason, glass is still commonly used for 2D culture on coverslips and slides. Drawbacks of glass are its fragility, which means that handling can be more difficult, as well as being more difficult to manufacture at small quantities, particularly in complex designs, which can lead to high costs for prototyping. Stainless steel can also be used for components of bioreactors and is commonly found in large-scale stirred tank systems due to its mechanical properties, chemical resistance and ease of cleaning making it suitable for repeated use [220]. For smaller bioreactor systems plastics are typically used instead of stainless steel due to their lower cost and ease of manufacturing, though some laboratories still use stainless steel for many components of their bioreactors [221].

Other issues can occur from materials that may seem on the surface to be ideal candidates for the creation of bioreactors. Commonly used sterilisation and disinfection procedures can cause changes to the chemical properties for a material potentially leading to unwanted biological effects [222,223]. For example, treatment of some polymers with laboratory disinfectant can lead to cytotoxicity from residual disinfectant absorbed into the polymer [224] whilst ultraviolet light (UV) treatment can also cause problems with some plastics, leading to changes in surface properties if exposed for periods of several hours [225]. Autoclaving generally leads to minimal increases in cytotoxicity and in some cases has even caused materials to have improved biocompatibility after autoclaving, however it is not compatible with plastics with low melting points such as conventional polystyrene cultureware [226]. Due to the wide range of available sterilisation methods, it is typically possible to find a method which is non-disruptive to the properties of the apparatus, allowing the preferred materials to be used.

### 4.2 Temperature variation and fluctuation

Cells and tissues require precise temperature control, typically at 37 °C, to maintain normal function and to promote reproducibility. Cell cultures are very sensitive to increases in media temperature and an increase of a few degrees can significantly reduce cell proliferation and viability over prolonged periods [227], [228], [229]. The effect of changing temperature goes beyond viability and proliferation, with many metabolic processes also affected. Other metabolic events can be altered by the changing temperature leading to inaccurate results in drug toxicity experiments [230] whilst expression of a range of proteins varies with the extent of the temperature increase [231].

The use of electronic equipment for powering perfusion in some bioreactor systems can impact the cultures through the effects it has on the temperature. With equipment that is thermally separated from the culture, for example power supplies which are outside the incubator or at a distance from the culture vessels, the impact of this is negligible. When equipment is in direct contact with the culture vessel, for example in the case of stirred or shaken bioreactors, undesirable additional heat can be transferred into the culture system. Whilst the impact of this is likely to only changed the temperature by a few degrees, as discussed in the previous paragraph this can have major repercussions over prolonged culture periods. As well as simply affecting cellular properties, this can also introduce variability between experiments, particularly if the contact regions have unequal heat distribution or if multi-culture systems are under different levels of load.

The impact of lower temperatures on cultures tends to be less heavily studied than increased temperatures but it has been shown to have effects such as a decreased metabolic rates and reduced proliferation [232,233]. Differentiation can also be altered with reduced temperature, with one study demonstrating effects such as delayed early differentiation and enlargement of granular cells in the epidermis of a full thickness skin model when cultured at temperatures of 33 °C and 35 °C [234]. The use of small fluid volumes or long lengths of tubing without adequate insulation or jacketing can lead to greater temperature fluctuation and reductions below the desired temperature. Whilst measurement of this effect is carried out infrequently, studies have shown that it can be done with relative ease to confirm that growth conditions are maintained at desirable levels for a range of system scales [45,235,236].

### 4.3 Evaporation and degradation of culture medium

The design and operation of a bioreactor system can have an impact on the quality of the culture medium over time. Long culture periods without media changes or use of small volumes can lead to high levels of evaporation which can be problematic for cell cultures, even in a humidified environment [237]. Large media volumes can be beneficial due to the more homogeneous media composition achieved without media changes. However, this can be impacted by evaporation and the resultant increase in the concentration of dissolved compounds over time. Use of media gas supplies such as oxygenators can also increase evaporation due to the addition of dry gas lowering the relative humidity, and bubble humidifiers are therefore often used [238,239]. Evaporative loss of medium still occurs in unsealed systems regardless of whether the environment is humidified. One study using a 24-well bioreactor system found an evaporation rate of roughly 0.6% per day over a 9-day culture period [103] whilst another study showed an evaporation rate of roughly 0.4 mL/day in a perfusion circuit at 20% relative humidity, whilst in the absence of humidity this was increased to around 1.4 mL/day [240].

Another potential issue with larger media volumes with minimal media changes aside from evaporation is the degradation of media constituents. Common media supplements such as L-glutamine and ascorbic acid are heat sensitive and therefore degrade over time in culture [241,242]. The degree to which this is a problem varies depending on specific conditions such as the culture period however it can also be very variable between cell types, with some cell lines being shown to be unaffected by L-glutamine degradation whilst others suffer a loss in viability [243]. This can be avoided by topping up the supplements periodically either directly or through partial media changes. In some cases supplements which are more stable can be used, for example in the case of L-glutamine a range of products using the L-alanyl-L-glutamine are available which have reduced build-up of ammonia compared to L-glutamine [244,245]. It is worth noting that not all supplements should be periodically topped up due to potential cytotoxic effects from large quantities of their degradation products [246,247].

Whilst replacement of the medium is beneficial for keeping nutrient and metabolic waste within desirable limits, it can also have undesirable effects on the function of cells. Over time in culture a variety of secreted molecules comprising the cell secretome influence the cellular function, and this communication has a key role for determining culture fate [248]. Secreted factors influence the viability, proliferation and function of cells, as well as being able to direct differentiation [249]. With pluripotent stem cells, for example, autocrine factors are secreted by the cells which aid with proliferation and maintenance of the undifferentiated state, whilst paracrine factors from other cell types have ability to lead embryonic stem cells down different pathways depending on the cells used for co-culture [250], [251], [252]. Whenever the medium is changed, these secreted factors are lost, and levels have to build back up in the fresh medium. Recirculation of medium can allow these factors to remain, whilst the use of conditioned medium also represents an alternative method to provide cells with necessary factors, even between media changes.

### 4.4 User interaction and manual handling

One problem which can occur in cell culture is the presence of biological contamination within the system. This can be in forms such as microbial contamination or cross-contamination with different cell lines [253], [254], [255]. Microbial contamination in cell culture, such as by bacteria or yeast, is an occasional occurrence in many laboratories and is often caused by poor aseptic technique during handling [256]. Even with the use of antibiotic and antimycotic agents it is still possible to get a contamination of these types, which negatively impact the experiments and lead to incorrect results, wasting time and money [257].

Careful aseptic technique is used to reduce the chances of contaminations and means that for the majority of the time these are not a problem during cell culture, however all handling of a sterile system increases the risk of contamination. Complex techniques and apparatus often require increased user interaction and therefore one challenge in the design of bioreactor systems is reducing the amount of handling of sterile components that is required to minimise the risk of contamination. Through careful optimisation of the design parameters, systems have been developed which can minimise these aspects of handling to maintain a high level of sterility [188].

One of the key uses of bioreactors for tissue engineering is to measure and control the conditions within the system, allowing fine tuning for tissues and cells of interest. Recent designs are frequently using continuous or non-invasive measurement systems to reduce the risk of handling cultures for data collection. Such probes can be maintained in the apparatus for the entire period and can be read through direct electrical connections or contactless methods. These can often be readily miniaturised and are now widely used in microfluidic culture systems for measurement of properties such as dissolved oxygen and pH [258], [259], [260].

As well as the contamination risk, manual handling can also have an impact on the reproducibility of the results. The reproducibility of published results is increasingly found to be a problem in cell biology and the creation of bioreactors which keep processes as simple as possible is key to generating systems in which results can be accurately reproduced between different individuals and laboratories [261], [262], [263]. Studies have shown that culture handling can be a potential source of error for assay results [264], and therefore minimal handling during ongoing culture is preferable to enhance the reproducibility of work and is something that should be factored into the design of bioreactors.

## 5 Limitations and future directions

While this review aimed to cover some of the major factors which influence the function and physiological relevance of bioreactor technology for the field of tissue engineering, there are many more properties which did not fit within the scope of this work. In a theoretical ‘perfect’ bioreactor system we might aim to control every environmental property which affect the cultures, with factors not mentioned here such as pressure and pH all at tightly controlled physiological levels whilst other factors such as oxygen might have their *in vivo* tissue oxygen gradients recreated. The reality is that with each additional factor controlled for, the subsequent complexity of the system increases and as such there is a trade-off between control and the time, cost and expertise required to develop and use the bioreactor.

A common concomitant of advancing system complexity is manual handling, increasing the contamination risk and disruption of the cell culture environment. While basic perfusion can be introduced rather simple with a rocker, more elaborate flow patterns may require pumps with adequate control units that can be space consuming and technically complex. High system complexity is also more prone to error, especially in long-term experiments that require steady and tightly controlled conditions. Each additional component of a system has to be compatible with the cell culture environment and is a possible source of variation which has to be considered and accounted for in experimental planning. Introduction of complex flow patterns also requires extensive testing to validate correct fluid flow within the system.

Further complexity is found through the interplay between different properties. Much like how mass transfer and shear stress are intrinsically linked to flow rate, so are other factors together such as carbon dioxide levels and the pH of the medium. These relationships make the creation of ideal conditions more difficult, with the movement of one towards more physiological levels having the potential to move the other further from them, and this is something that has not been covered in this review. In such cases it is often that a compromise must be found which promotes the physiological function of cells as best as possible within the limits of the system. New culture platforms are being created at great speed to push these boundaries and ongoing technological advances are further supporting the capacity for replication of physiological conditions and monitoring these in real time. Non-invasive monitoring of factors such as pH, glucose and oxygen concentration in the medium can give a real-time view of conditions over an entire culture period [265] whilst new sensors are being developed to allow a wider range of proteins to be continually monitored [266,267]. Highly penetrative imaging using multiphoton microscopy can allow detailed analysis of thick tissue equivalents and can be minimally invasive through the use of methods like second harmonic generation, which can allow label-free visualisation of proteins including collagen I [268,269].

Another potential limitation is the difficulty determining the ideal fluid flow conditions for a given organ due to the complexity of tissue structure. As a prime example, in the liver the commonly used cells for modelling drug metabolism *in vitro* are hepatocytes. These cells experience low levels of shear stress due to the protective effect of the sinusoidal endothelial cells however also have high levels of mass transfer due to the highly permeable, fenestrated surfaces of the sinusoidal endothelial cells [270]. These low shear stress conditions are significantly different to the experience of sinusoidal endothelial cells, as well as Kupffer cells, the resident liver macrophages, which reside within the sinusoidal lumen [271]. The variability is further complicated by the nutrient gradient present in the liver lobule, with periportal cells experiencing high levels of oxygenation whilst pericentral cells experience it at reduced levels, a factor which helps to determine the specific cell function [272]. These cell-specific conditions are key to accurately recapitulating a tissue *in vitro*, however, also introduce significant technical challenges. Studies often develop models for studying a specific physiological condition and can therefore minimise the complexity in terms of number of cell types and spatial organisation. This makes it easier to provide biomimetic conditions to the cells that are included however can limit the transferability of models between different applications.

## 6 Conclusion

The development of perfusion bioreactors is a fast progressing field and new systems being produced for a wide range of different applications the requirement for characterising the properties of systems in as much depth as possible is increasingly important to ensure reproducibility and applicability of results between systems [263]. In a perfusion bioreactor a key aspect is the properties of the fluid flow. With properties such as flow rate, shear stress and fluid mixing all having significant impacts on the health of tissues these need to be tailored or the tissue of interest. Modelling of the fluid flow is a powerful tool for both characterisation and development of a system and is now routinely carried out for newly developed bioreactor systems [273], [274], [275], [276]. The ability to accurately model the flow in a system computationally without the need for time intensive prototyping and testing can speed up the initial stages of design and allow issues to be picked up early in the development process.

A series of other properties can impact the tissue health. Factors such as cytotoxicity of materials are universal to the design of any system and therefore care should be taken to select materials which are most appropriate and effective for the requirements of a system. Other factors tend to be specific to particular methods being utilised such as evaporation of media, temperature fluctuations and media degradation which are of concern for systems with low volumes or long culture periods. Meanwhile, some properties are far more subjective and need to be weighed against the criteria for the system. High levels of user interaction can have an impact on the success and reproducibility of culture techniques, and therefore minimising the need for interaction where possible is generally preferable. While some of these parameters may be of less importance in the early development stages of a bioreactor, reproducibility, ease of use and cost should generally be regarded as key aims for systems which are aiming for wider adoption.

The factors considered herein aim to cover a range of possible requirements for benchtop perfusion bioreactor systems. The range of applications for these systems coupled with individual constraints such as readily available equipment and space means that there is no one-size-fits-all approach to creating perfusion bioreactors for tissue engineering. There is also a range of properties found *in vivo* which can be controlled on top of those dealt with specifically by bioreactor systems and can have an impact on cell and tissue culture outcomes such as medium composition, heterocellularity and cellular patterning. These are all factors which also need to be taken into account when designing the optimum conditions to encompass the huge complexity of physiologically tissue microenvironment. By discussing these key factors, and examining the complex interactions between them, this review aims to promote the future development of benchtop bioreactor systems for culturing tissue equivalents. A reduction in development cost, both financially and in time spent on optimisation, through incorporating these properties in the early stages of the design process will support more novel bioreactor technology to reach routine use in laboratory experiments. Further to this, with the wide range of different bioreactor systems in use, thorough understanding and reporting of culture conditions can improve comparability between studies and boost the research impact of future studies.

## Conflict of interest

Author S.P. is affiliated with the company Reprocell Europe. All other authors declare no competing interests.
